# Assessing the external validity of the VALIDATE-SWEDEHEART trial

**DOI:** 10.1177/17407745211012438

**Published:** 2021-05-20

**Authors:** Rebecca T Rylance, Philippe Wagner, Elmir Omerovic, Claes Held, Stefan James, Sasha Koul, David Erlinge

**Affiliations:** 1 Department of Cardiology, Clinical Sciences, Lund University and Skåne University Hospital, Lund, Sweden; 2 Center for Clinical Research, Uppsala University, Västerås, Sweden; 3 Department of Molecular and Clinical Medicine, Institute of Medicine, Sahlgrenska University Hospital, Gothenburg, Sweden; 4 Department of Cardiology, Sahlgrenska University Hospital, Gothenburg, Sweden; 5 Department of Medical Sciences and Cardiology, Uppsala Clinical Research Center, Uppsala University, Uppsala, Sweden; 6 Swedish Society of Cardiology, Uppsala, Sweden

**Keywords:** VALIDATE-SWEDEHEART, nationwide registry data, real-world, anticoagulants, percutaneous coronary intervention

## Abstract

**Aims:**

The VALIDATE-SWEDEHEART trial was a registry-based randomized trial comparing bivalirudin and heparin in patients with acute myocardial infarction undergoing percutaneous coronary intervention. It showed no differences in mortality at 30 or 180 days. This study examines how well the trial population results may generalize to the population of all screened patients with fulfilled inclusion criteria in regard to mortality at 30 and 180 days.

**Methods:**

The standardized difference in the mean propensity score for trial inclusion between trial population and the screened not-enrolled with fulfilled inclusion criteria was calculated as a metric of similarity. Propensity scores were then used in an inverse-probability weighted Cox regression analysis using the trial population only to estimate the difference in mortality as it would have been had the trial included all screened patients with fulfilled inclusion criteria. Patients who were very likely to be included were weighted down and those who had a very low probability of being in the trial were weighted up.

**Results:**

The propensity score difference was 0.61. There were no significant differences in mortality between bivalirudin and heparin in the inverse-probability weighted analysis (hazard ratio 1.11, 95% confidence interval (0.73, 1.68)) at 30 days or 180 days (hazard ratio 0.98, 95% confidence interval (0.70, 1.36)).

**Conclusion:**

The propensity score difference demonstrated that the screened not-enrolled with fulfilled inclusion criteria and trial population were not similar. The inverse-probability weighted analysis showed no significant differences in mortality. From this, we conclude that the VALIDATE results may be generalized to the screened not-enrolled with fulfilled inclusion criteria.

## Introduction

The results of randomized controlled trials are usually the basis on which medications are approved for public use. Efficacy trials primarily use a homogeneous set of participants to ascertain whether an intervention is successful under optimal conditions.^1^ Primary focus is on internal validity, that is, the legitimacy of the study in terms of design and limiting bias.^2^ Less attention has been given to external validity,^3^ which refers to the extent to which results of a randomized trial can be generalized to a real-world population.

The VALIDATE-SWEDEHEART trial was a registry-based randomized trial comparing bivalirudin and heparin for patients who underwent percutaneous coronary intervention without planned glycoprotein IIb/IIIa inhibitors after acute myocardial infarction.^4^ It showed no differences in the composite endpoint at 180 days, which included death from any cause, acute myocardial infarction or bleeding. It showed no differences in the secondary endpoint of death either, which is the endpoint of focus for this study. The external validity of VALIDATE should be further examined as VALIDATE had an open-label design in which participating physicians may have been biased, and the trial population may not have been representative of all patients undergoing percutaneous coronary intervention. There are no accepted guidelines for evaluating external validity in randomized controlled trials,^5^ and many conditions may affect external validity, including the trial setting as compared with routine practice, patient selection, the characteristics of the trial participants, differences between the trial protocol and clinical practice, and the inclusion and exclusion criteria.^6^

The external validity of trials related to acute myocardial infarction, percutaneous coronary intervention, and antithrombotic medications has not been studied in patients who fulfilled inclusion criteria upon screening, but were not enrolled in the trial. The SWEDEHEART registry provides a unique opportunity to conduct such research. We investigated the difference between the cases with fulfilled inclusion criteria but not enrolled (hereafter referred to as ‘screened not-enrolled’) and the trial population in terms of 30 and 180 days mortality to assess the correspondence of results to those of the VALIDATE-SWEDEHEART trial. In addition, we investigated what the trial results would have been, had it included all screened patients with fulfilled inclusion criteria.

## Methods

### Trial population

The VALIDATE-SWEDEHEART (bivalirudin versus heparin in ST-Segment and Non-ST-Segment Elevation Myocardial Infarction Registry Trial) was a multicentre, controlled, registry-based randomized clinical trial comparing treatment effects between bivalirudin and heparin in conjunction with the percutaneous coronary intervention for acute myocardial infarction.^4,7,8^ A composite of death from any-cause, acute myocardial infarction and bleeding at 180 days was the primary endpoint. Twenty-five of Sweden’s 29 percutaneous coronary intervention centres participated in the trial. Of the 12,561 patients screened for participation, 6555 were not randomized and 6006 (48%) were randomized and referred to in this article as the trial population.

### Screened not-enrolled

Of the 6555 non-randomized patients, 150 had a missing treatment assignment in the registry and were excluded, leaving 6405 in the screened not-enrolled population, a secondary population of interest in this study. Of those 6405, 3422 did not meet the inclusion criteria for various reasons: not able to give informed consent (22%), not treated with ticagrelor or prasugrel (21%), indicated as other reason for non-inclusion in the registry (19%), 5000 U of heparin before arrival or 3000 U in the lab before angiography (13%), not ST-segment elevation myocardial infarction/non-ST-segment elevation myocardial infarction (9%), reduced kidney function (5%), planned glycoprotein IIb/IIIa inhibitors (3%), life expectancy of 1 year or less (3%), contraindication for either heparin or bivalirudin in the trial (2%), continuous bleeding (2%), uncontrolled hypertension, thrombocytopenia, and endocarditis (0.9%), and not being 18 years old (0.1%). Thus, 2983 remained as the screened not-enrolled population with fulfilled inclusion criteria. The reasons these cases were not randomized were not documented. The VALIDATE-SWEDEHEART trial was powered for evaluation of ST-segment elevation myocardial infarction and non-ST-segment elevation myocardial infarction separately with enrolment of an equal number of each type of acute coronary syndrome. Thus, 3001 patients with non-ST-segment elevation myocardial infarction and 3005 with ST-segment elevation myocardial infarction were enrolled. In the general SWEDEHEART population, however, there were more cases with non-ST-segment elevation myocardial infarction than ST-segment elevation myocardial infarction, which made the population of screened not-enrolled cases even more unbalanced with respect to the numbers with non-ST-segment elevation versus ST-segment elevation. In addition, some of the hospitals did not include patients during off-hours, that is, night shifts or the weekends, and some physicians did not participate in the study.

### The study populations

**Figure 1.**
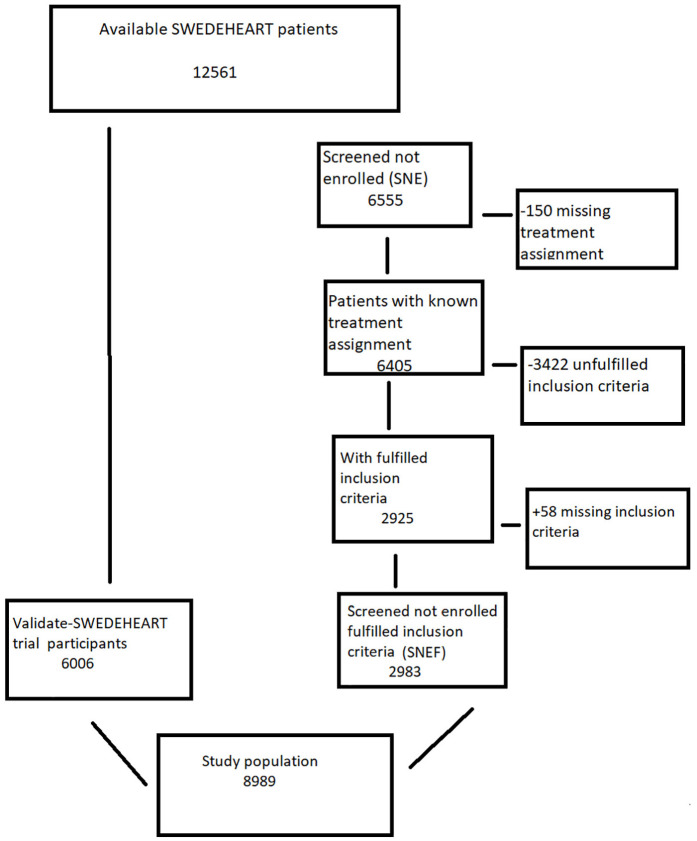
Flowchart.

The two patient populations were combined into a single dataset for further analysis, 6006 randomized in the VALIDATE trial and 2983 with known treatment assignment and satisfying inclusion criteria. Those patients who did not meet the inclusion criteria were removed, leaving the screened not-enrolled with fulfilled inclusion criteria (Figure 1).^9^ Thus, in total, 8989 patients were included in this study. Since patients were not randomized to bivalirudin or heparin, these treatment groups were not balanced with respect to the observed covariates (Table 1). An additional analysis of all 12,411 screened patients with known treatment assignments was also performed (Supplementary Material). We used death at 30 and 180 days as the primary endpoint.

**Table 1.** Baseline table of trial population and screened not-enrolled with fulfilled inclusion criteria.

Characteristics	TP (n = 6006)	SNEF(n = 2983)	TP Bivalirudin(n = 3004)	TP Heparin(n = 3002)	SNEFBivalirudin(n = 853)	SNEF Heparin(n = 2130)
STEMI, n (%)	3005 (50.0)	1049 (35.2)*	1501 (50.0)	1504 (50.1)	567 (66.5)	482 (22.6)**
Male sex, n (%)	4406 (73.4)	2092 (70.1)*	2229 (74.2)	2177 (72.5)	601 (70.5)	1491 (70.0)
Age Median, year	68.0	70.0*	68.0	68.0	67.0	70.0*
IQR, year	60.0–75.0	60.0–78.0	59.0–75.0	60.0–75.0	57.0–76.0	62.0–78.0
≥65, n (%)	3671 (61.1)	1963(65.8)	1819 (60.6)	1852 (61.7)	494 (57.9)	1469 (69.0)
BMI Median	26.9	26.9	26.8	26.9	27.0	26.9
IQR	24.5–29.7	24.3–30.1	24.5–29.7	24.5–29.7	24.2–30.2	24.3–30.1
Weight < 60 kg, n (%)	295 (4.9)	209 (7.0)*	139 (4.6)	156 (5.2)	68 (8.0)	141 (6.6)
Previous Smoker, n (%)	2047 (34.1)	1032 (34.6)*	1027 (34.2)	1020 (34.0)	270 (31.7)	762 (35.8) **
Current smoker, n (%)	1426 (23.7)	650 (21.8)*	716 (23.8)	710 (23.7)	218 (25.6)	432 (20.3)**
Diabetes, n (%)	999 (16.6)	672 (22.5)*	491 (16.3)	508 (16.9)	154 (18.1)	518 (24.3)**
Hypertension, n (%)	3105 (51.7)	1728 (57.9)*	1557 (51.8)	1548 (51.6)	426 (49.9)	1302 (61.3)**
Hyperlipidaemia, n (%)	1889 (31.5)	1195 (40.1)*	953 (31.7)	936 (31.2)	271 (31.8)	924 (43.4)**
Previous AMI, n (%)	974 (16.2)	739 (24.8)*	490 (16.3)	484 (16.1)	168 (19.7)	571 (26.8)**
Previous PCI, n (%)	882 (14.7)	593 (19.9)*	456 (15.2)	426 (14.2)	123 (14.4)	470 (22.1)**
Previous CABG, n (%)	293 (4.9)	268 (9.0)*	152 (5.1)	141 (4.7)	52 (6.1)	216 (10.1)**
Previous stroke, n (%)	240 (4.0)	145 (4.9)*	115 (3.8)	125 (4.2)	38 (4.5)	107 (5.0)**
CPR, n (%)	46 (0.8)	33 (1.1)*	26 (0.9)	20 (0.7)	14 (1.6)	19 (0.9)**
Killip class II, III and IV, n (%)	194 (3.2)	169 (5.7)*	108 (3.6)	86 (2.9)	84 (9.9)	85 (4.0)**
Puncture site femoral vein, n (%)	570 (9.5)	565 (18.9)*	290 (9.7)	280 (9.3)	204 (23.9)	361 (17.0)**
Ace inhibitor on admission, n (%)	990 (16.5)	586 (19.6)*	501 (16.7)	489 (16.3)	139 (16.3)	447 (21.0)**
Off hours, n (%)	576 (9.6)	226 (7.6)*	288 (9.6)	288 (9.6)	23 (2.7)	203 (9.5)**
Creatinine-median (micromole/L) IQR	80 (68–93)	80 (68–95)*	80 (68–93)	79 (68–92)	79 (66–94)	80 (68–95)**

AMI: acute myocardial infarction; PCI: percutaneous cornoary intervention; CABG: previous coronary artery bypass graft; CPR: cardiopulmonary resuscitation; TP: trial population; SNEF: screened not-enrolled with fulfilled inclusion criteria.

* Significant differences between TP & SNEF.

** Significant differences between bivalirudin and heparin in SNEF.

## Methods

**Figure 2.**
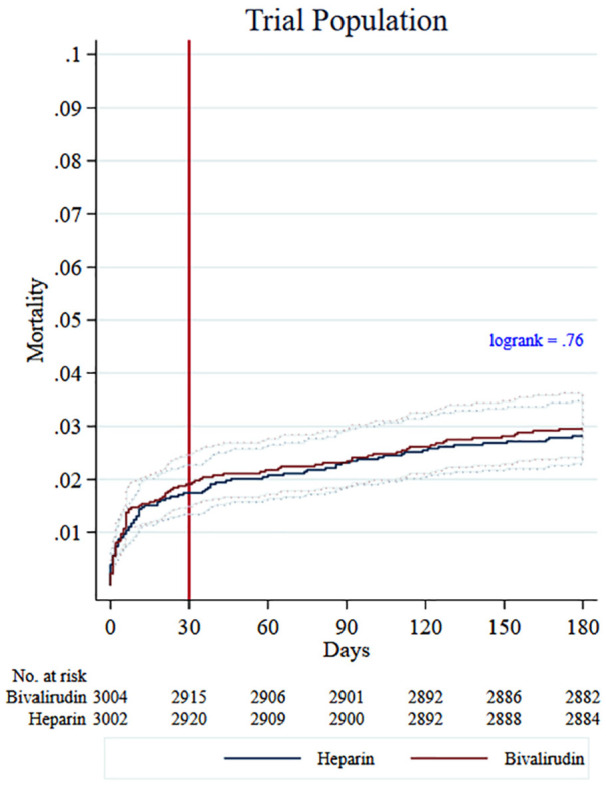
Kaplan Meier failure curves trial population.

**Figure 3.**
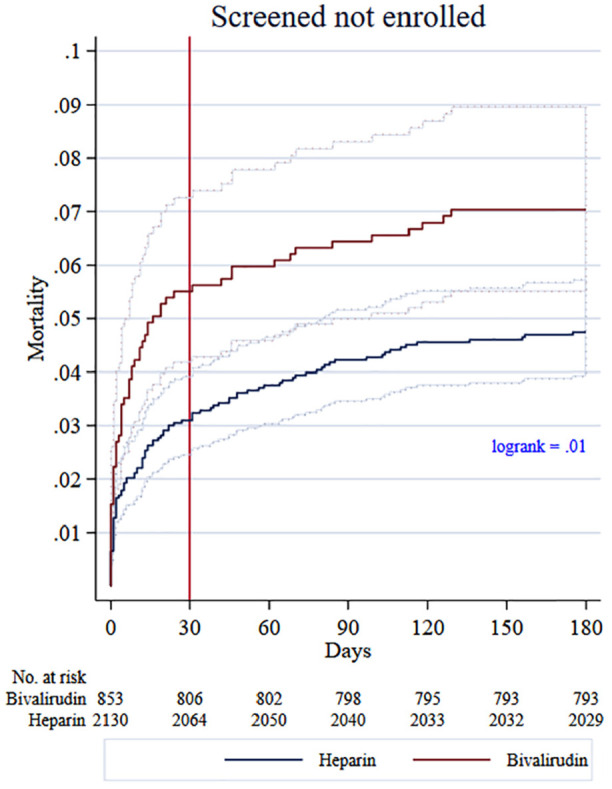
Kaplan Meier failure curves screened not-enrolled with fulfilled inclusion criteria population.

A baseline table, Kaplan–Meier curves, and log-rank test were produced to describe the study data (Table 1, Figures 2 and 3). A propensity score method, using logistic regression with inclusion to VALIDATE as the outcome to generate scores, was used to evaluate population similarity with respect to prognostic factors between the trial population and the screened not-enrolled population.^9^ This was done in a stratified analysis of the cases with and without ST-segment elevation as well. The variables chosen for the propensity score analysis were from the original publication.^4^ Traditionally, propensity scores are used to mimic randomized trials through balancing observed covariates between treatment groups in observational studies.^10^ The lower the propensity score difference, the more similar the populations.^8^ Logistic regression modelled the probability of being selected in the VALIDATE trial with variables of interest. These covariates were potentially associated with differences in VALIDATE trial selection. Additional variables were included to ensure balance.^11^ The model included the following variables: ST-segment elevation myocardial infarction / non-ST-segment elevation myocardial infarction, age, sex, low weight, smoking, diabetes, hypertension, hyperlipidaemia, previous acute myocardial infarction, previous percutaneous coronary intervention, previous coronary artery bypass graft, previous stroke, renal failure, thrombectomy, ticagrelor before inclusion, clopidogrel before inclusion, cardiopulmonary resuscitation, puncture location, creatinine (µmol/L), angiotensin-converting enzyme inhibitors, Killip class and off-hours versus regular hours (Supplementary Table 5). Off-hours signified the night or weekend shifts. Incidence proportions for off-hours and regular hours were calculated for the different groups and endpoints. Categorical variables that had between 2% and 4% missing were altered to include an extra category for missing; these variables were smoking, cardiopulmonary resuscitation, angiotensin-converting enzyme inhibitors, previous stroke, and off-hours versus regular hours. Killip class had 12% missing and was treated similarly by adding an extra category for missing. However, all models were performed with the Killip class variable with an extra category for missing and the original variable without manipulation. A complete case approach was implemented for all models after data alterations for missingness.

Propensity scores were then used in an inverse-probability weighted Cox regression analysis using the trial population to estimate the difference in mortality as it would have been had the trial included all screened patients with fulfilled inclusion criteria.^7^ The point of the weights is to give greater weight to patients with characteristics similar to those screened but not enrolled.^12^ These weights were checked via trimmed and stabilized weighting.^13^ All the variables that were included in the inverse-probability weighted analysis were checked in separate Cox models as part of an interaction term with the treatment variable. A sensitivity analysis solely including the significant variables from this subgroup analysis were produced to see if the mean propensity score difference changed.

For the inverse-probability weighted analysis and generalization to be valid, it is important that the trial and not-enrolled populations are not substantially different, and while the standardized mean difference in propensity scores measured similarity in measured prognostic factors, equally important are differences with respect to other, unmeasured factors. To examine such issues, we investigated differences in adjusted mortality rates between the trial and not-enrolled populations in the same treatment arms. That is, we compared mortality between those on bivalirudin in the trial with those on bivalirudin in the not-enrolled population, and analogously, those on heparin in the trial with those on heparin in the not-enrolled population, adjusted for measured prognostic factors. If differences between populations were detected, we concluded that there may be differences in unmeasured prognostic factors between populations. The analysis was performed by means of a Cox regression model including a *treatment × trial selection* interaction term. The interaction term helped create the needed hazard ratio’s comparing mortality across treatments, that is, heparin treatment in the screened not-enrolled with fulfilled inclusion criteria compared with heparin treatment in the trial population. The same was produced for bivalirudin versus bivalirudin. In addition, as a by-product, it produced a hazard ratio for the treatment effect in the screened not-enrolled population divided by the corresponding hazard ratio in the trial population. However, the comparison of bivalirudin and heparin in the not-enrolled population does not aid in the generalization of the trial results to a larger population.

The analysis was adjusted for: ST-segment elevation myocardial infarction/ non-ST-segment elevation myocardial infarction, age, gender, low weight, smoking, diabetes, hypertension, hyperlipidaemia, previous acute myocardial infarction, previous percutaneous coronary intervention, coronary artery bypass graft, previous stroke, renal failure, thrombectomy, ticagrelor before trial, clopidogrel before trial, cardiopulmonary resuscitation, puncture location, creatinine (µmol/L), angiotensin-converting enzyme inhibitors, Killip class and regular versus off-hours. We refer to this analysis as the adjusted Cox model (Tables 2 and 3). These analyses of adjusted differences in mortality were performed as a means of identifying potential differences unaccounted for by measured covariates. Knowledge of such is paramount when evaluating differences between populations and generalizing results. These analyses were repeated as a sensitivity analysis with separate models for ST-segment elevation myocardial infarction/ non-ST-segment elevation myocardial infarction populations to see if the treatment effects changed by type of myocardial infarction. All analyses were performed in STATA/MP version 15. All tests were two-sided and the alpha-level was set to 0.05. Hazard ratios are presented with 95% confidence intervals (CI).

**Table 2.** Adjusted model with interaction (Treatment and Inclusion to Validate) mortality at 30 days.

TP and SNEFinteractionmodel	TP event %BivalirudinHeparin, HRBivalirudinvs. Heparin *	SNEF event %BivalirudinHeparin,HR Bivalirudinvs. Heparin*	SNEF HR Bivalirudinvs. Heparin	SNEF vs. TP Ratio of HR Bivalirudinvs. Heparin	SNEF vs. TPHR Heparin vs.Heparin andHR Bivalirudinvs. Bivalirudin
All patients n = 8989	1.9% vs. 1.7% HR 1.10 CI (0.75 1.59) P = 0.635	5.5% vs. 3.1% HR 1.80 CI (1.24 2.62) P = 0.002	HR 1.00 CI (0.63 1.60) P = 0.991	HR 0.91 CI (0.49 1.68) P = 0.760	HR 1.54 CI (1.01 2.36) P = 0.046 HR 1.40 CI (0.89 2.22) P = 0.150
STEMI n = 4054	2.9% vs. 2.7% HR 1.08 CI (0.70 1.66) P = 0.734	6.9% vs. 7.9% HR 0.87 CI (0.56 1.36) P = 0.534	HR 0.84 CI (0.48 1.46) P = 0.526	HR 0.87 CI (0.42 1.80) P = 0.698	HR 1.50 CI (0.88 2.56) P = 0.132 HR 1.30 CI (0.77 2.21) P = 0.328
NSTEMI N = 4935	0.9% vs. 0.8% HR 1.16 CI (0.54 2.51) P = 0.703	2.8% vs. 1.7% HR 1.65 CI (0.75 3.63) P = 0.210	HR 1.71 CI (0.72 4.05) P = 0.223	HR 1.24 CI (0.39 4.01) P = 0.716	HR 1.73 CI (0.83 3.60) P = 0.146 HR 2.14 CI (.84 5.48) P = 0.112

TP: trial population; SNEF: screened not-enrolled with fullfiled inclusion criteria; HR: hazard ratio; CI: 95% confidence interval; P: p-value.

* Interaction term only (Treatment and Inclusion to Validate).

**Table 3.** Adjusted model with interaction (Treatment and Inclusion to Validate) mortality at 180 days.

TP and SNEFInteractionmodel	TP event%BivalirudinHeparin,HR Bivalirudin vs.Heparin *	SNEF event%BivalirudinHeparin,HR Bivalirudin vs.Heparin *	SNEF HR Bivalirudinvs. Heparin	SNEF vs. TPRatio of HRBivalirudinvs. Heparin	SNEF vs. TPHR Heparinvs. Heparinand HR Bivalirudinvs. Bivalirudin
All patients n = 8989	3.0% vs. 2.8% HR 1.05 CI (0.78 1.41) P = 0.762	7.0% vs. 4.7% HR 1.51 CI (1.10 2.08) P = 0.012	HR 1.02 CI (0.70 1.50) P = 0.913	HR 1.02 CI (0.62 1.70) P = 0.912	HR 1.31 CI (0.94 1.83) P = 0.110 HR 1.35 CI (0.91 1.98) P = 0.126
STEMI n = 4054	3.9% vs. 3.9% HR 1.00 CI (0.70 1.44) P = 0.984	8.8% vs. 9.8% HR 0.90 CI (0.60 1.34) P = 0.596	HR 0.92 CI (0.57 1.50) P = 0.731	HR 1.05 CI (0.56 1.97) P = 0.868	HR 1.30 CI (0.83 2.05) P = 0.254 HR 1.37 CI (0.87 2.15) P = 0.168
NSTEMI N = 4935	2.0% vs. 1.7% HR 1.15 CI (0.68 1.94) P = 0.606	3.5% vs. 3.3% HR 1.07 CI (0.55 2.10) P = 0.841	HR 1.08 CI (0.52 2.23) P = 0.832	HR 0.92 CI (0.37 2.29) P = 0.862	HR 1.41 CI (0.85 2.33) P = 0.185 HR 1.30 CI (.60 2.83) P = 0.512

TP: trial population; SNEF: screened not-enrolled with fullfiled inclusion criteria; HR: hazard ratio; CI: 95% confidence interval; P: p-value.

* Interaction term only (Treatment and Inclusion to Validate).

## Results

### Inclusion in VALIDATE analysis

The standardized propensity score difference of being included to VALIDATE was 0.61. When including only significant variables from the sensitivity subgroup analysis of variables that interact with treatment, the propensity score difference was reduced to 0.42. The significant variables were ST-segment elevation myocardial infarction/ non-ST-segment elevation myocardial infarction, age, coronary artery bypass graft, previous stroke, renal failure, puncture site, and creatinine level. When the differences were stratified by type of acute myocardial infarction, the results were 0.51 for ST-segment elevation myocardial infarction and 0.58 for non-ST-segment elevation myocardial infarction.

### Mortality at 30 days

The incidence of all-cause mortality was 1.8% in the trial population, 3.8% in the screened not-enrolled population, and 5.2% in the population including those that did not fulfil eligibility criteria. The incidence of death for off-hours and regular hours was, respectively, 2.4% and 1.4%, 5.1% and 3.0%, and 7.8% and 3.7% in these populations. Mortality rates with bivalirudin and heparin treatment are set out in Table 2. The log-rank test showed significant differences in the survival curves for the screened not-enrolled population in favour of heparin, (P = .01), but not in the trial population, (P = 0.76) (Figures 2 and 3). For the trial population, there were no significant differences in mortality for either patients with ST-segment elevation myocardial infarction or non-ST-segment elevation myocardial infarction. The results in the screened not-enrolled population were also similar for ST-segment elevation myocardial infarction and non-ST-segment elevation myocardial infarction (Supplementary Material).

There were no significant differences in mortality between treatment groups in the inverse-probability weighted analysis of the trial population, which was re-weighted to be closely aligned to the pooled trial and screened not-enrolled populations (hazard ratio 1.11, 95% CI (0.73, 1.68)). The adjusted Cox model revealed no significant differences in mortality between bivalirudin and heparin in the screened not-enrolled population (hazard ratio 1.00, 95% CI (0.63, 1.60)). There were no significant differences in the hazard ratio for bivalirudin versus heparin between the two populations (hazard ratio 0.91, 95% CI (0.49, 1.68)) (Table 2). The Cox model showed significantly higher mortality for the screened not-enrolled population compared with the trial population for cases treated with heparin (hazard ratio 1.54, 95% CI (1.01, 2.36)), but not for those treated with bivalirudin (hazard ratio 1.40, 95% CI (0.89, 2.22)). The differences with only ST-segment elevation myocardial infarction or only non-ST-segment elevation myocardial infarction were all non-significant (Table 2). The factors that predicted death at 30 days were ST-segment elevation myocardial infarction, age, sex, smoking, diabetes, renal failure, thrombectomy, ticagrelor before inclusion, puncture site, angiotensin-converting enzyme inhibitors, creatinine and Killip class.

Results for mortality at 180 days, shown in Table 3, demonstrated broadly similar trends to the preceding analyses at 30 days.

## Discussion

The current study compared the results from a registry-randomized trial to real-world data. We found significant differences in the unadjusted failure curves in the screened not-enrolled cases at 30 days, with heparin being advantageous over bivalirudin. This could be explained by the fact that heparin was more frequently administered to less vulnerable patients.^14^ These differences were greatly attenuated, however, in the adjusted models.

The screened not-enrolled group was sicker than the trial population, which may be attributed to selection bias on the part of the investigators not choosing to randomize sicker patients. A difference in prognostic factors was observed. There were, however, no differences between bivalirudin and heparin in the inverse-probability weighted analysis, which may indicate that these differences do not affect the generalization of trial results to all screened patients with fulfilled inclusion criteria. The fact that they were sicker was additionally confirmed by results from the adjusted Cox model that showed increased mortality in the not-enrolled group, even after adjustment for known prognostic factors, indicating possible differences in unobserved prognostic factors.

Additional analysis of the non-randomized populations have not been performed for other SWEDEHEART registry-randomized controlled trials like DETO2X^15^ and TASTE,^16^ so this type of study is a novel addition to SWEDEHEART in the registry-randomized controlled trial setting. These types of studies are a possibility for DETO2X^15^ and TASTE^16^as a complimentary addition to gain a better understanding of how results may change in other populations.

An observational SWEDEHEART study^17^ that included patients from January 2007 to December 2014 and all percutaneous coronary intervention conducted in Sweden found significantly higher 30-day mortality in the heparin group in the adjusted analysis but not in the propensity-matched analysis.^17^ The VALIDATE-SWEDEHEART trial found no differences between bivalirudin and heparin at 30 days. The source of the discrepancy observed in this previous trial compared with VALIDATE-SWEDEHEART may be the inclusion of patients from an earlier time-span and centres lacking experience with bivalirudin. The advantage of our study is that it is more recent with the same time-span as the VALIDATE-SWEDEHEART study, and staff at all included hospitals were experienced in providing both tested treatments, which may eliminate some bias.

Outside of SWEDEHEART, the MATRIX trial found significant differences in mortality between bivalirudin and heparin at 30 days in favour of bivalirudin for all-cause mortality as well as cardiac causes in ST-segment elevation myocardial infarction and non-ST-segment elevation myocardial infarction patients, but they allowed planned glycoprotein IIb/IIIa inhibitors in the heparin group.^18,19^ The HORIZONS-AMI trial also found bivalirudin to be superior to heparin and glycoprotein IIb/IIIa inhibitors for death and cardiac specific causes of death at 30 days in stable coronary artery disease and non-ST-segment elevation myocardial infarction patients.^20^ The HEAT-percutaneous coronary intervention study included 97% of available ST-segment elevation myocardial infarction patients thereby encompassing a broad spectrum of patients with delayed consent approval. There were no significant differences between bivalirudin and heparin with bailout glycoprotein IIb/IIIa inhibitors for death at 28 days.^21^ One could argue that the results from HEAT-percutaneous coronary intervention are based on a representative sample of real-world data albeit only ST-segment elevation myocardial infarction patients, which makes a direct comparison to the previous two studies difficult. It does however make it comparable to the ST-segment elevation myocardial infarction patients in both the screened not-enrolled with fulfilled inclusion criteria and the screened not-enrolled analysis in this study. We can conclude that the results of both these analysis for ST-segment elevation myocardial infarction patients coincide with the results from the HEAT- percutaneous coronary intervention trial of no differences between bivalirudin and heparin.

Studies on real-world data are paramount as an addition to randomized trials, although there is no consensus on the meaning of the term real-world data.^22^ A carotid artery study based on the National Cardiovascular Database Registry–Carotid Artery Revascularization and Endarterectomy (NCDR) Registry found large discrepancies between participants in the postmarketing study and those who were not included.^23^ Using the NCDR Cath Percutaneous Coronary Intervention Registry, a comparison of patients’ baseline demographics and outcomes who were included in the dual antiplatelet therapy participating hospitals and those who were not was performed. Those who were not included in dual antiplatelet therapy had on average significantly longer hospital stays and multi-vessel disease.^24^

## Conclusions

Even though observational studies are complicated in terms of design and confounders, they provide vital information.^25^ The current study contributes to the literature on real-world data and echoes concerns that those who are included in randomized studies are not a representative sample of all patients, even in a registry setting. This study aimed to address the question of generalizability of the VALIDATE-SWEDHEART trial and indicated that there would still be no difference found between the bivalirudin and heparin treatment arms, had those screened but not enrolled been included in the study.

## Supplemental Material

sj-pdf-1-ctj-10.1177_17407745211012438 – Supplemental material for Assessing the external validity of the VALIDATE-SWEDEHEART trial Click here for additional data file. Supplemental material, sj-pdf-1-ctj-10.1177_17407745211012438 for Assessing the external validity of the VALIDATE-SWEDEHEART trial by Rebecca T Rylance, Philippe Wagner, Elmir Omerovic, Claes Held, Stefan James, Sasha Koul and David Erlinge in Clinical Trials
