# Supplementary material for: Assessing the external validity of the VALIDATE-SWEDEHEART trial
Source: Clin Trials. 2021 May 20;18(4):427–35. doi: 10.1177/17407745211012438 (PMC8290983; doi:10.1177/17407745211012438)
Supplement: sj-pdf-1-ctj-10.1177_17407745211012438 – Supplemental material for Assessing the external validity of the VALIDATE-SWEDEHEART trial [file sj-pdf-1-ctj-10.1177_17407745211012438.pdf]

Appendix

Kaplan-Meier

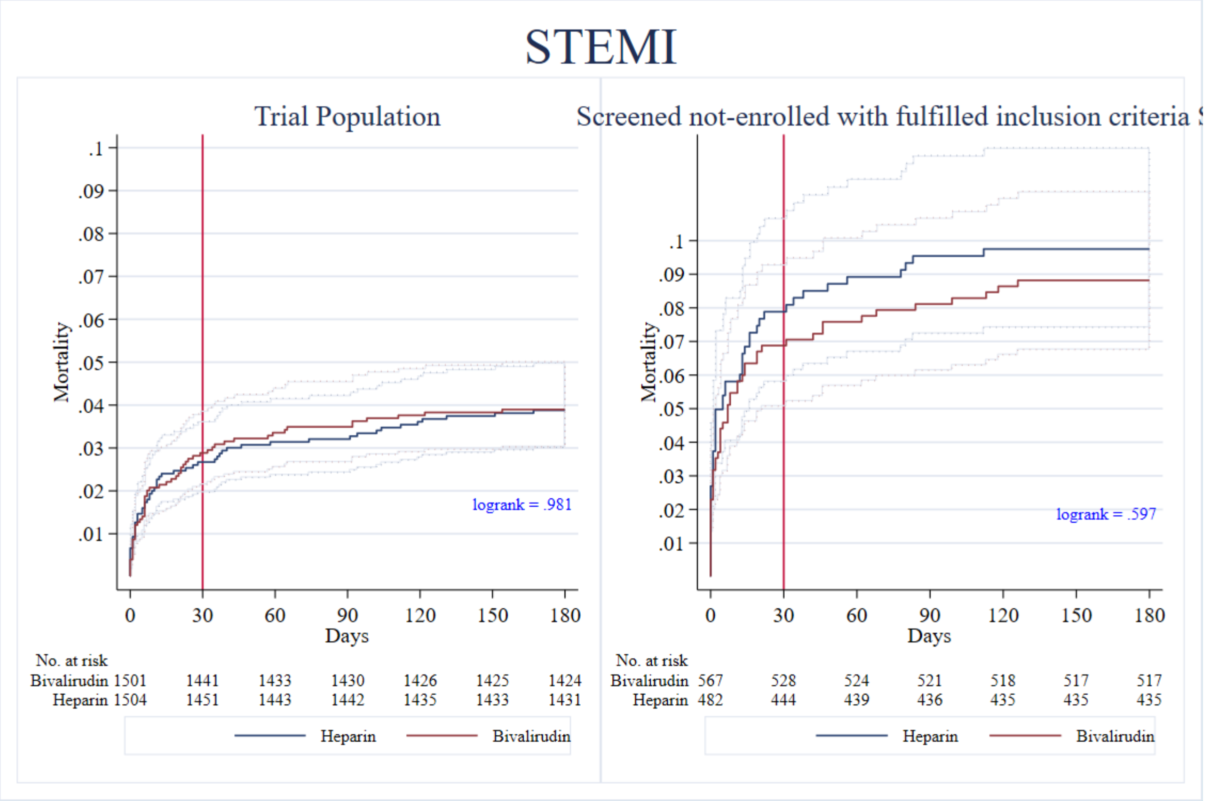

STEMI- ST-segment elevation myocardial infarction

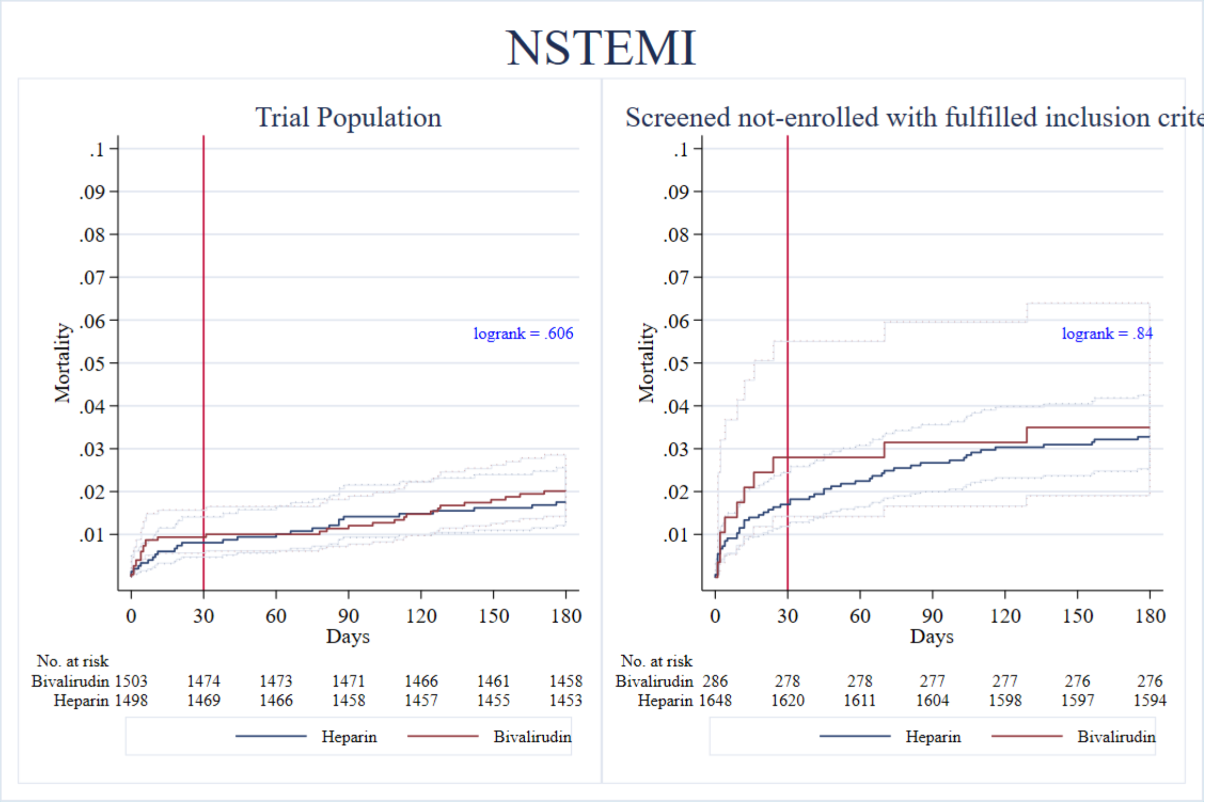

NSTEMI- non-ST-segment elevation myocardial infarction

Supplemental Table 1. Adjusted model with interaction (Treatment and Inclusion to Validate) mortality at 30 days for all screened patients

TP and SNE Interaction model	TP event % Bivalirudin Heparin, HR Bivalirudin vs. Heparin *	SNE event % Bivalirudin Heparin, HR Bivalirudin vs. Heparin *	SNE HR Bivalirudin vs. Heparin	SNE vs. TP Ratio of HR Bivalirudin vs. Heparin	SNE vs. TP HR Heparin vs. Heparin and HR Bivalirudin vs. Bivalirudin
All patients n= 12411	1.9% vs. 1.7% HR 1.10 CI (0.75 1.60) P = 0.635	7.2% vs. 4.6% HR 1.57 CI (1.25 1.98) P < 0.001	HR 0.92 CI (0.69 1.23) P = 0.584	HR 0.87 CI (0.53 1.43) P = 0.584	HR 1.57 CI (1.11 2.23) P = 0.010 HR 1.37 CI (0.94 2.00) P = 0.103
STEMI n=5242	2.9% vs. 2.7% HR 1.08 CI (0.70 1.66)	8.3% vs. 11.4% HR 0.71 CI (0.54 0.93)	HR 0.78 CI (0.55 1.09) P = 0.146	HR 0.79 CI (0.438 1.42) P = 0.426	HR 1.54 CI (1.01 2.34) P = 0.045

	P = 0.734	P = 0.013			HR 1.21<br>CI (0.78 1.89)<br>P = 0.401
NSTEMI<br>N=7169	0.9% vs. 0.8%<br>HR 1.16<br>CI (0.54 2.51)<br>P = 0.703	4.7% vs. 2.5%<br>HR 1.93<br>CI (1.20 3.09)<br>P = 0.007	HR 1.39<br>CI (0.78 2.48)<br>P = 0.259	HR 1.04<br>CI (0.39 2.77)<br>P = 0.939	HR 1.91<br>CI (1.00 3.66)<br>P = 0.051<br>HR 1.98<br>CI (0.94 4.20)<br>P = 0.073

TP-Trial population
SNE-Screened not enrolled
*** Interaction term only (Treatment and Inclusion to Validate)**
HR - Hazard ratio
CI - 95% Confidence interval
P - p-value

Supplemental Table 2. Adjusted model with interaction (Treatment and Inclusion to Validate) mortality at 180 days for all screened patients

TP and SNE Interaction model	TP event % Bivalirudin Heparin, HR Bivalirudin vs. Heparin *	SNE event % Bivalirudin Heparin, HR Bivalirudin vs. Heparin *	SNE HR Bivalirudin vs. Heparin	SNE vs. TP Ratio of HR Bivalirudin vs. Heparin	SNE vs. TP HR Heparin vs. Heparin and HR Bivalirudin vs. Bivalirudin
All patients<br>n= 12411	3.0% vs. 2.8%<br>HR 1.05<br>CI (0.78 1.41)<br>P = 0.762	9.8% vs. 7.4%<br>HR 1.36<br>CI (1.12 1.64)<br>P = 0.002	HR 0.96<br>CI (0.75 1.21)<br>P = 0.714	HR 0.97<br>CI (0.71 1.34)<br>P = 0.856	HR 1.58<br>CI (1.21 2.06)<br>P = 0.001<br>HR 1.56<br>CI (1.14 2.11)<br>P = 0.005
STEMI<br>n=5242	3.9% vs. 3.9%<br>HR 1.00<br>CI (0.70 1.44)<br>p=0.984	11.4% vs. 15.5%<br>HR 0.72<br>CI (0.57 0.90)<br>P = 0.005	HR 0.86<br>CI (0.65 1.14)<br>P = 0.303	HR 0.95<br>CI (0.58 1.55)<br>P = 0.835	HR 1.65<br>CI (1.17 2.33)<br>P = 0.004<br>HR 1.57<br>CI (1.08 2.28)<br>P = 0.017
NSTEMI<br>N=7169	2.0% vs. 1.7%<br>HR 1.15<br>CI (0.68 1.94)<br>P = 0.606	6.2% vs. 4.8%<br>HR 1.32<br>CI (0.89 1.97)<br>P = 0.168	HR 1.23<br>CI (0.80 1.95)<br>P = 0.371	HR 1.10<br>CI (0.54 2.25)<br>P = 0.783	HR 1.59<br>CI (1.02 2.46)<br>P = 0.039<br>HR 1.75<br>CI (0.99 3.11)<br>P = 0.055

TP-Trial Population
SNE- Screened not enrolled
*** Interaction term only (Treatment and Inclusion to Validate)**
HR - Hazard ratio
CI - 95% Confidence interval
P - p-value

Supplemental Table 3.

Stratified mean difference SNE

**Inclusion into VALIDATE**	0.70
STEMI	0.65
NSTEMI	0.66

Supplemental Table 4.

IPW analysis of SNE

At 30-days	HR 1.02, CI (0.61 1.72)].
At 180-days	HR 0.88, CI (0.55 1.41)]

Supplemental Table 5. Logistic Regression - Inclusion to Validate outcome SNEF & TP

	Validate Odds Ratio	P	CI
Diabetes	0.83	0.004	0.73,0.94
Gender	0.92	0.144	0.81,1.03
STEMI	1.38	<0.001	1.24,1.55
Age	1.00	0.257	0.99,1.00
Low weight	0.73	0.003	0.59,0.90
Smoking	0.96	0.172	0.91,1.02

Status			
Hypertension	0.95	0.364	0.85,1.06
Hyperlipidaemia	0.97	0.662	0.85,1.11
Previous AMI	0.69	<0.001	0.57,0.83
Previous PCI	1.12	0.222	0.93,1.35
Previous CABG	1.00	0.971	0.81,1.24
Previous Stroke	1.29	0.003	1.09,1.53
Renal failure	0.98	0.801	0.83,1.15
Trombectomy	0.87	0.231	0.70,1.09
Previous Ticagrelor	1.15	0.036	1.01,1.32
Previous Clopidogrel	0.59	<0.001	0.47,0.75
Killipklass	0.61	<0.001	0.57,0.65
CPR	0.71	<0.001	0.60,0.85
Puncture site	0.52	<0.001	0.45,0.61
ACE Inhibitors	1.03	0.577	0.92,1.16
Creatinine	1.00	0.156	1.00,1.00
Off hours	1.26	<0.001	1.15,1.39
Constant	10.67	<0.001	6.86,16.59
N	8493		
